# Experiences of sexual health in persons with hip and knee osteoarthritis: a qualitative study

**DOI:** 10.1186/s12891-020-03596-5

**Published:** 2020-08-24

**Authors:** Emma Nilsing Strid, Marie Ekelius-Hamping

**Affiliations:** 1 University Health Care Research Center, Faculty of Medicine and Health, Örebro University, Örebro, Sweden; 2 Primary Health Care Southern Division, Region Örebro County, Sweden

**Keywords:** Osteoarthritis, Sexual health, Physical therapists, Qualitative research, Pain

## Abstract

**Background:**

Osteoarthritis (OA) is the world’s most common form of arthritis and a common cause of musculoskeletal pain and disability. Persons living with chronic diseases often have affected sexual health because of pain and limited function. Osteoarthritis is a chronic disease but there is scarce knowledge about how sexual health is experienced. The aim of this study was to explore the experience of sexual health in persons who have seen a physiotherapist for their hip and/or knee OA.

**Methods:**

This study has a qualitative design. Participants were recruited from the Swedish Quality Register Better Management of Patients with Osteoarthritis (BOA). To reach a variety of experiences and strengthen credibility, a purposeful sampling strategy based on age, sex and hip and knee OA was used. Semi-structured telephone interviews were held with 20 persons with hip and/or knee OA. Data were analysed with qualitative content analysis and inductive category development was applied.

**Results:**

The analysis resulted in two main categories. The first category, Individual differences in how sexual health is affected by hip and knee OA, comprises of two sub categories: Pain limits sexual health; and Strategies for sexual health in the relationship. The second main category, Varying needs for communication about sexual health, is supported by the sub categories: Physiotherapists do not ask about sexual health; and Relevance of communicating about sexual health.

**Conclusions:**

Painful hip and knee OA limit sexual health to varying degrees, and individuals make adjustments or develop strategies to maintain sexual life. Sexual health is not talked about during consultations with physiotherapists or other health care professionals, indicating that patients with OA may have unmet needs regarding their sexual health. Further research is needed on how to provide support and information about sexual health in OA.

## Background

Osteoarthritis (OA) is the eleventh cause of disability in the world, causing significant health and economic burden [1, 2]. It is a chronic degenerative joint disease and may develop in any joint, but is most frequent in hip, knee, spine, fingers and toes [1–3]. The incidence increases with age and rates of OA are higher among women [2, 3]. Because of the growing age of the population and the increase in obesity and sedentary lifestyle throughout the world, it is anticipated that the number of people living with OA will increase and become a major problem for health systems globally [1]. Hip and knee OA is a common cause of musculoskeletal pain, stiffness and muscle weakness, all of which may lead to activity limitations, such as difficulties in walking, carrying objects and dressing, as well as reduced quality of life [1–3]. An important domain of quality of life is sexual health, defined by the World Health Organization (WHO) as –.

… a state of physical, emotional, mental and social well-being in relation to sexuality; it is not merely the absence of disease, dysfunction or infirmity. Sexual health requires a positive and respectful approach to sexuality and sexual relationships, as well as the possibility of having pleasurable and safe sexual experiences, free of coercion, discrimination and violence. For sexual health to be attained and maintained, the sexual rights of all persons must be respected, protected and fulfilled [4].

Previous research has reported high prevalence of impaired sexual health among persons living with chronic diseases such as rheumatoid arthritis (RA) [5–9], fibromyalgia [10, 11] and multiple sclerosis [12]. Sexual dysfunction is also reported to be higher among persons with RA compared with healthy control groups, and the main influencing factor is pain [13, 14]. Restoux et al. (2020) concluded, in a recent systematic review based on 50 quantitative and six qualitative studies, that sexual dysfunction is highly prevalent among both men and women with inflammatory arthritis, and called for increased clinician awareness of this impairment, so as to guide provision of tailored education and support [15]. Increased awareness is important especially since previous studies have highlighted that the majority of health care professionals do not proactively discuss sexuality issues with patients [16, 17]. Among the perceived barriers are worry about causing offense, personal discomfort, lack of time, resources or knowledge which can be represented in a model including organizational, structural and personal factors [16].

As OA is a chronic joint disease, it can be expected that sexual health might be affected, but there is scarce scientific knowledge. Previous research has primarily assessed sexual health before and after total hip arthroplasty [18–20] or knee arthroplasty [21]. Approximately 45% of those with knee OA [21] and 64–82% of those with hip OA who will undergo arthroplasty report impaired sexual health [18, 19]. However, the expectations of sexual activity after surgery are not always fulfilled [18]. Experiences of living with hip and knee OA, and how it affects the person’s life at many levels, have previously been explored [22–24], but, to the authors’ knowledge, without addressing sexual health or communication with physiotherapists. The aim of this study was to explore the experience of sexual health in persons who have seen a physiotherapist for their hip and/or knee OA.

## Methods

This study has a qualitative explorative design based on data collected through individual semi-structured telephone interviews with persons with hip and/or knee OA. An explorative study design can provide insight into a research area that has not previously been investigated and interviews may give a rich understanding of participants’ experiences expressed in their own words [25].

### Setting and sampling strategy

**Fig. 1:**
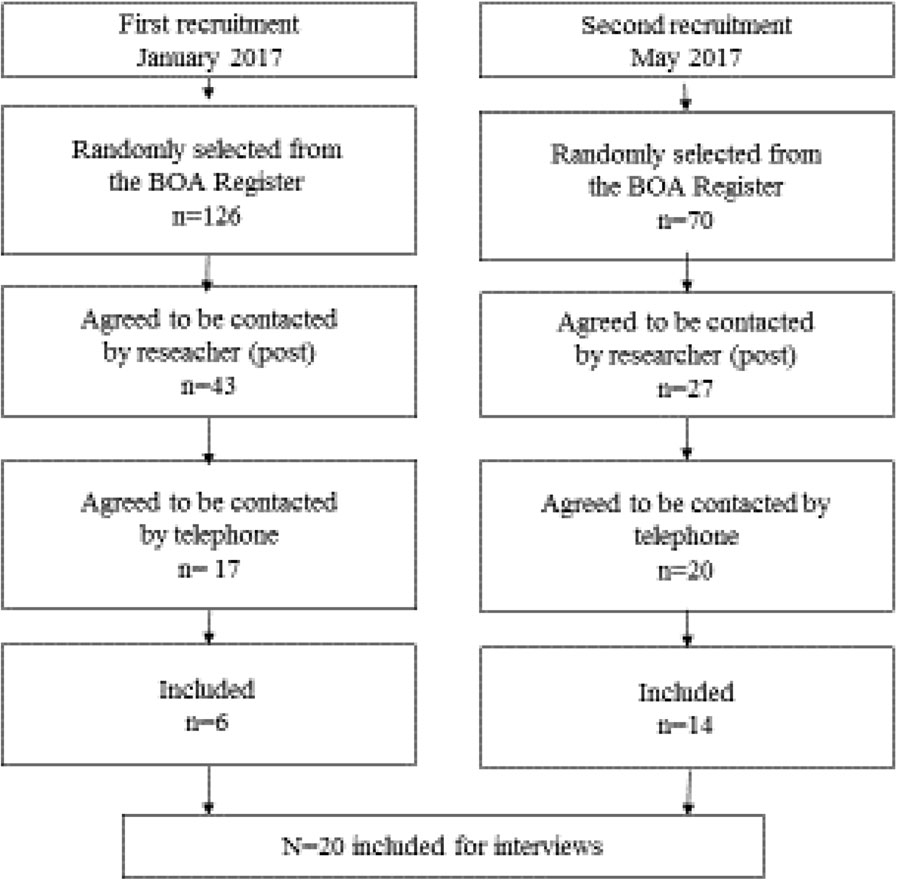
Flow chart of the sampling process

Participants were recruited from the Swedish National Quality Register for patients with OA, Better Management of Patients with Osteoarthritis (BOA), a register comprising patient-reported outcomes collected before and after a supported OA self-management program in primary care [26] . Patients who seek care for hip, knee or hand pain with clinical determined and/or radiographic diagnosis of OA can be referred by a physiotherapist to the program. In 2017 there were approximately 90,000 persons registered with BOA nationwide. Inclusion criteria for this study were: hip and/or knee OA; and living with someone. Exclusion criteria were: joint arthroplasty in hip and/or knee; difficulties walking for reasons other than hip and/or knee pain; and being registered at the clinic where the physiotherapist responsible for conducting the interviews (M.E.H.) was working, since she held the educational sessions in the OA program and therefore met all OA patients at the clinic. To obtain a variety of experiences, the sample strived to be heterogeneous regarding sex, age and OA in hip and knee. In January 2017, 126 potential participants were randomly selected from the BOA register across a specified set of demographic and clinical characteristics as stated above (Fig. 1). Brief information about the study and the request for permission for contact details to be forwarded to the researcher was sent by post from the BOA Register administration. Forty-three persons, who returned written consent to be further contacted, were sent extended written information about the study and conditions for participation. Twenty persons answered and 17 gave their written consent to be contacted, which was done in January–May 2017. Six of these 17 persons were included: eight were excluded because of surgery to the hip or knee; two did not wish to participate; and one could not be reached.

To enhance sample size and data quality, a second recruitment was performed in May 2017 following the same procedure as for the first recruitment. A purposeful sample of 70 persons were randomly selected from the BOA Register. Twenty-seven persons who returned a written informed consent to be contacted, were sent extended information about the study and conditions for participation. Twenty-one persons answered and 20 gave their written consent to be contacted, which was done in September 2017–May 2018. Five persons were excluded because of surgery to either the hip or the knee, and one person could not be reached. Fourteen persons remained for interview in this second recruitment.

### Data collection

A semi-structured interview guide was developed based on previous research and discussions among the authors and research colleagues. A pilot interview was conducted to evaluate the questions and fine-tune interview techniques and procedures. Because of the revisions made, the pilot interview was not included in the final analysis. The final interview guide included questions about how hip and knee OA affects daily life and sexual health, and about communication with the physiotherapist regarding sexual health. Probing questions were used to develop and deepen the answers.

Semi-structured interviews were held by telephone in February 2017–May 2018. The interviewer (M.E.H.) had no former relationship with any of the participants. Time for the interview was set in agreement with each participant. Oral information about the study, including conditions for participation, and the opportunity to ask questions were provided before each interview. Each interview lasted between 19 and 31 min. The interviews were digitally recorded and transcribed verbatim by a professional transcriber or by the interviewer (M.E.H.). The transcripts were reviewed for accuracy and matched with the audio files. The transcripts were then imported to NVivo 11, which was used to manage and code the data (QSR International, Melbourne, Australia). The interviewees were pseudonymized and each assigned a number from 1 to 20.

### Data analysis

The data analysis was performed after all the interviews had been held. Participants were recruited until the sample size was judged to yield maximum variation. In this study, informational redundancy was deemed to have been reached since the last interviews did not provide any relevant new information. Qualitative content analysis and an inductive category development was used [25]. Only the visible and obvious in the text, i.e. the manifest meaning of the text, was analysed according to the aim of the study, which was to describe the participants’ experiences from their own perspective.

Initially, every transcript was read thoroughly to get a sense of each participant’s story and, next, a sense of the whole by reading all transcripts. During additional reading and by the use of Nvivo, statements in the text (meaning units) responding to the study aim were identified. These meaning units were coded as close as possible to the text and provided with a label describing each individual statement. These codes were compared and contrasted and then merged into subcategories and, finally, categories based on similarities and differences. The content within each category had to be internally homogenous while separate categories should be externally heterogeneous. Tentative categories were modified and redefined, and new categories were developed when needed. This analytical process involved working back and forth between the categories and text to verify the meaningfulness and accuracy of the categories [25].

The first transcript was independently analysed by the two authors and the meaning units, codes and a tentative coding tree was discussed. The analysis was then performed by M.E.H. and continuous discussions on consistency of codes, final subcategories and categories were held with E.N.S. throughout the analysis process until agreement was reached. Quotes capturing the essence of the interviews were selected to illustrate the different subcategories. The selected quotes from the transcript were translated into English and then retranslated into Swedish, to ensure that the meaning was retained.

### Ethical considerations

Permission to conduct the study was approved by the regional ethical board in Uppsala, Sweden (No. 2016/242). The participants provided written informed consent after receiving both written and oral information about the study, including assurance of the voluntary nature of participation and the possibility to withdraw at any time without explanation and, further, assurance that all collected data would be handled confidentially and no individual would be identifiable in the quotes or results. Only the authors had access to the original audio files, transcripts and informed consents. The data and the code key were stored separately and locked away safely. The participants were informed of the interviewer’s professional background, and that the interview data would be analysed, and published in a research journal. The study followed the Consolidated Criteria for Reporting Qualitative Research (COREQ) checklist [27].

## Results

In total, 20 persons were finally interviewed in the study, 13 women and seven men. The participants were between 52 and 77 years old, eleven had knee OA, six had hip OA and three had both knee and hip OA. Two categories emerged that described the participants’ experiences of sexual health: Individual differences in how sexual health is affected by hip and knee OA, and Varying needs for communication about sexual health. The findings are presented in four subcategories developed in the analysis (Table 1). Quotes from the participants are presented in the text to illustrate each of the subcategories.

**Table 1:** Main categories and subcategories describing experiences of sexual health in persons with hip and knee osteoarthritis (OA)

Category	Subcategories	
Individual differences in how sexual health is affected by hip and knee OA	Pain limits sexual health	Strategies for sexual health in the relationship
Varying needs for communication about sexual health	Physiotherapists do not ask about sexual health	Relevance of communicating about sexual health

### Individual differences in how sexual health is affected by hip and knee osteoarthritis

The first main category highlights a variation in the extent to which persons with OA of the hip and/or knee experience that their sexual health is affected by OA. The category consists of two subcategories: Pain limits sexual health; and Strategies for sexual health in the relationship.

#### Pain limits sexual health

The participants’ experiences ranged from having impaired sexual health and feeling limited in their sexual life because of OA, which could evoke negative emotions, to having unaffected sexual life despite pain and stiffness. The vast majority of the participants described that OA in hip and/or knee led to pain during sexual activity and hindered them from being fully sexually active, despite a desire to continue as previously. Some described that their sexual life was not affected at all as they only had knee OA, but when OA also occurred in the hip it became more difficult. Impaired hip mobility made it too painful and affected sexual life more than did the reduced mobility in the knee joints. In the quotation below, a participant describes situations during intercourse when the affected joint ends up at a painful angle because of the reduced joint mobility.

*No – it’s that I kind of don’t manage to get my legs up, so to speak. Didn’t manage to press my legs together, if I might say so. Kind of like – yeah, and it doesn’t feel particularly comfortable then. I know about it – almost as if I’m waiting for it – ouch! ouch! Like that, you know? (I15)*

Expressions of resignation over having a sexual desire but being limited by high pain intensity, and sadness over the loss of previous spontaneity were related. Some said that whether they could be intimate or have intercourse or not was dependent on what activities they had been doing that day and the day before and how much pain this had generated. Sexual desire may remain despite both pain and stiffness, but sexual activities must then be adapted, which can limit sexual health.

*Yeah, of course we’d like to be able to be a bit more spontaneous … whenever we happen to be in the mood … but it does affect things a bit more, mm-hm. (I4)*

The participants related that their sexual life could be almost unaffected during times of less pain. There were also a few descriptions of a completely unaffected sexual life despite pain and restricted mobility, where no changes in either sexual frequency or otherwise had to be made because of the OA. However, high pain intensity or constant pain limited sexual life both during and after intercourse. Those who had described more pain previously or who described episodic pain, also reported that pain limited sexual life more during the painful times.

*It has limited me in certain positions – that it has. When I was in the most pain, I was of course – I mean we did have – our sex life certainly has, I mean it certainly has been affected – has definitely created a few limitations, but nothing that I’ve ... um ... neither I nor my wife have felt that it’s been any kind of impediment. (I12)*

Experience of pain during intercourse could lead to negative thoughts, fear of pain and avoidance of sexual activities. The participants also described pain at the beginning of intercourse, but said that it felt better afterwards, which is in line with how they experienced symptoms related to OA in physical activities. The importance of sexual life was emphasized and pain during or after intercourse was described as “worth it”; that is, the pain intensity was perceived as acceptable and the pain quickly passed.

*Yeah, it’s more afterwards, actually. During the activity I’d say you don’t think about that so much. (I4)*

#### Strategies for sexual health in the relationship

All the participants were living with a partner, which was one of the inclusion criteria. Their experiences included making adjustments and developing different strategies, either by themselves or in conjunction with their partner, to maintain sexual life despite pain and other impairments due to OA. Making adjustments to maintain sexual life was described with acceptance by some; others had more difficulties making adjustments and expressed a desire to change positions during intercourse even though they knew it would not work on account of their painful hip or knee. The persons gave examples of preferred positions as well as difficulties with some positions such as lying on top, or spreading the legs wide apart, and said that they needed a soft surface under the knees.

*Yes, if we say that ... when we start to have intercourse, I’ll want to lie on my back ... yeah, that’s what I preferred. Yes ... that was best for me because that way I relaxed my leg. But, like I said ... uh ... no problems now, that I can say. (I13)*

Some of those living in a long-term relationship described that they talked to each other and tried to find solutions together. Others did not communicate about sex; instead, they handled the sexual issues in the moment but pointed out the importance of their partner showing understanding for the pain and being careful during intercourse.

*No, but we simply had to try different things and find out what worked. If you have a – how should I say – a long-term, open relationship, obviously you talk about it, too. (I13)*

Some described that they had tried to maintain sexual life for a long time, but then had had to stop having sexual intercourse because it had become too difficult or painful. This decision had resulted in feelings of calmness but also sadness. Other ways to be intimate that did not include intercourse were described as important, such as caresses, hugs, kisses and cuddles.

*So things have gotten calmer for both of us ... now that we don’t really have any sex life; like that. (I20)*

### Varying needs for communication about sexual health

The second main category consists of the two subcategories: Physiotherapists do not ask about sexual health; and Relevance of communicating about sexual health.

#### Physiotherapists do not ask about sexual health

None of the participants had ever been in a consultation where the physiotherapist or any other health care professional had brought up sexual health or asked if they wanted to discuss their sexual health related to hip and/or knee OA, even though some participants expressed a need for discussing sexual health with their health professional. The need to talk about sexual health varied among the participants. Some expressed a need to discuss sexual health, but they had not thought of physiotherapists as a conversation partner for this. Descriptions of difficulties for them as patients to raise the topic of sex and issues of their sexual life also emerged. Health care visits are usually limited to physical symptoms and limitations during daily activities, and this is what physiotherapists normally look at. So it was difficult as a patient to bring up sexual health in contacts with health care, as described by a participant in the quotation below.

*No, and perhaps it doesn’t come easy for everyone to talk about it, either ... that’s the way it is – it’s a bit taboo, unfortunately. (I7)*

To not have been asked about their sexual health by physiotherapists was easily excused, as it may not be easy to address the issue of sexual health with patients of higher age. These preconceptions may also make it even more difficult for the participants as patients to ask questions about sexual health; in their view, the social norm was that sexual life for many older persons has ceased because of age.

*No – why might that be? Perhaps it’s because many of us who have osteoarthritis are elderly and then maybe society de-emphasizes sexuality for the elderly, without asking, so to speak. (I6)*

#### Relevance of communicating about sexual health

Opinions differed about whether physiotherapists were the right health care professionals to communicate with about sexual health. Those participants who were more hesitant questioned whether physiotherapists have the right education and whether they are competent to discuss sexual health with their patients. They therefore found it surprising that physiotherapists could be approached regarding sexual health issues. Others described physiotherapists as an excellent professional group for communicating with about issues concerning sexual health. Some wanted to receive advice regarding sexual health in addition to exercises and other advice, as expressed in the quotation below.

*Well, no doubt I would have found it quite considerate. To think, “Oh my goodness! Am I actually going to get help with that as well?” (I5)*

Trust and confidence in the physiotherapist was described as an important prerequisite for bringing up issues regarding sexual health. Participants suggested that information on sexual health in relation to hip and knee OA could be provided in OA groups or in the supported OA self-management program. It was suggested to generally raise the issue at these, and that personal questions could be discussed afterwards individually with a physiotherapist. The OA self-management program include men and women of different ages, and some described that they were not comfortable talking about sexual health in this mixed group.

Another suggestion was that a brochure with information and advice on sexual health, including intercourse positions, be made available for OA patients.

*No, but that ... I would think it would be great to have, in an osteoarthritis program – plain and simple, I mean, and then you might find yourself thinking that if there’s something you need you could ask a question about it. (I16)*

## Discussion

The main finding of this study reveals that painful hip and knee OA affects sexual health to varying degrees. The participants described adjustments and strategies to maintain their sexual life despite pain related to OA. The issue of sexual health had not been addressed in their contact with physiotherapists or other health care professionals, indicating that patients with OA may have unmet needs regarding their sexual health. Prior survey studies have reported that most of those who undergo hip and knee replacement surgery are sexually active, but the prevalence of impaired sexual health is high [18–21]. To the best of our knowledge, no previous studies have explored OA patients’ experiences of sexual health. One advantage of qualitative research is the possibility to describe and understand the complexity of a patient’s situation [25]. Because the research on sexual health in OA patient populations is scarce, we will below discuss the findings of this study compared with previous research on sexual health in other patient populations, such as RA and fibromyalgia patients. Persons with RA and fibromyalgia have affected sexual health due to pain and reduced joint mobility, but other symptoms such as fatigue, cognitive dysfunction, sleep disorders, and depression may also have an impact on their sexual health [5, 7, 11], symptoms that may also be prevalent among OA patients.

To feel bothersome pain before, during and after intercourse can affect sexual life, as described by the participants in this study as well in another study with RA patients [7]. The pain experience could lead to negative feelings such as fear of pain, which in turn could lead to avoidance of sexual activities comprising the intimate relationship. Fear of increased pain and difficulties to find good positions during intercourse are also described by persons with fibromyalgia, RA and chronic pain [7, 10, 28]. In this study, the participants described a lot of “trial and error” intercourse and that their daily functioning determined whether sexual activity was possible at all. They expressed sadness over this loss of spontaneity.

There were some participants in this study who said that their sexual health was not affected at all. The extent to which sexual health is affected by OA may be related to the impact of the disease and ordinary circumstances in life, but it may also reflect the dynamics of individual relationships [15]. It may also be in line with the findings from a previous qualitative study in persons with RA who described that their sexual life was impaired when RA first appeared, but over the years they had found ways of adapting to their new situation [29]. The participants in our study described adjustments and strategies to cope with their impairments and maintain sexual health, with or without intercourse. This was often achieved through open communication with their partner. A variety of strategies were described, such as changing or choosing certain positions during intercourse and interrupting sexual intercourse and replacing it with caresses or other ways to express intimacy. These strategies have previously been acknowledged as self-management strategies [29, 30]. The adjustments were made and strategies adopted with varying degrees of acceptance and evoked emotions of calmness but also resignation and sadness, which in itself may impair sexual health. Sexual health is an important part of quality of life, including those with chronic diseases and poor health, which points to the fact that these issues need to be addressed [4, 31].

Since sexual dysfunction appears to be highly prevalent among both men and women with inflammatory arthritis [15] and OA [18–21], increased clinician awareness of this impairment is needed to guide provision of tailored information and support [32]. In the present study, as well as in earlier research [8, 29, 30, 33], patients have stated that health care professionals do not include sexual health in their assessment or interventions without prompting. Patients may be unaware of the impact of the disease on their sexual function or assume that no help is available [34]. None of the participants in the present study had been asked about their sexual health by their physiotherapist or another health care professional; neither had any of them initiated conversations about their sexuality with their health professional. This finding is in line with previous research suggesting that sexuality may still be a sensitive and taboo subject in the health care system [33, 34], which was specifically expressed by participants in the current study. Even though individual needs vary, the opportunity to discuss sexual health should be given by health care professionals. As suggested by Traumer, only by routinely addressing sexual health with patients diagnosed with chronic diseases can this taboo be broken and thereby signal to patients that it is acceptable and safe to discuss sexual health with health care [33]. In a recent editorial, health care professionals are encouraged to consider assessment of sexual health and implement strategies to optimize sexual health as an integrated part of their management [32].

The majority of the participants in this study stated that they would value sexual health being addressed within the health care system, such as information provided in a brochure or as part of the information provided by physiotherapist in the supported OA self-management program. The participants emphasized the importance of having confidence and trust in the health care professional before discussing sexual health, and some suggested physiotherapists as an excellent conversation partner. At times, even if physiotherapists could play an important role as a promoter for sexual health, they may in general not have the required knowledge to address sexual health [35, 36]. Further studies are needed to investigate whether and how sexual health should be routinely addressed by physiotherapists within OA assessment and interventions, and reported in the BOA National Quality Register.

### Methodological considerations

This study employed qualitative content analysis to explore experiences of sexual health in persons with hip and/or knee OA. Qualitative findings from information-rich samples can be applied to other samples with similar characteristics under similar conditions [25]. This study focused on experiences of persons with hip and/or knee OA in an attempt to learn more about how OA affects sexual health. The purposeful sample from the BOA Register provided a nationwide variation regarding sex, age, and hip and knee OA, which enables the reader to judge whether the findings are transferable to other populations with knee or hip OA. A study limitation regarding transferability is that we did not collect data on participants’ functioning or scores of OA severity. The inclusion criteria “to live with someone” used to select participants from the BOA register may not infer that the participant is in sexual relationship. However, the included participants were living with a partner with whom they had an intimate relationship with and they were willing to discuss sexual health. Twenty persons out of 196 eligible were finally interviewed, which may reflect a recruitment difficulty due to the subject being sensitive and taboo. We have no information of those who did not want to participate.

The interviews were held over the phone, allowing recruitment of participants from across Sweden. In addition to decreased costs and increased reach of geographically disparate participants, telephone interviews may allow participants to feel relaxed and speak freely and disclose intimate or sensitive information, but without evidence of producing lower data quality such as lower depth [37]. Both authors are women and experienced physiotherapists working in primary healthcare, M.E.H. has excellent knowledge about OA and E.N.S is a qualified qualitative researcher. Continuing discussions were held between the authors during data collection to enhance interview techniques. To deal with the notion that the findings may be shaped according to clinical or personal biases, a systematic inductive category development was performed which involves looking for alternative explanations (Patton 2015). Also, consensus discussions were held among the authors during the analysis and the final categories were discussed at a scientific seminar. Trustworthiness was determined by following rigorous methodology for inclusion, data collection and analysis, but included no member check. The checklist for reporting qualitative studies was used to improve transferability [27].

## Conclusions

Painful hip and knee OA limits sexual health to varying degrees, and individuals make adjustments or develop strategies to maintain sexual life. Sexual health is not normally addressed during a consultation with the physiotherapist or other health care professional, indicating that patients with OA may have unmet needs regarding their sexual health. Further research is needed on how to provide support and information about sexual health in OA.

## Supplementary information

**Additional file 1.** Interview guide.

## Data Availability

The data generated and analysed during the current study are governed by the legal provisions of Region Örebro County, Sweden. The datasets generated and analysed during the current study are not publicly available owing to the sensitive and personal nature of the data, according to Swedish Data Protection Law, but will be available from the corresponding author on reasonable request.

## Abbreviations

BOA: Better Management of Patients with Osteoarthritis
OA: Osteoarthritis
RA: Rheumatoid arthritis

## References

[1] Cross M, Smith E, Hoy D, Nolte S, Ackerman I, Fransen M (2014). The global burden of hip and knee osteoarthritis: estimates from the global burden of disease 2010 study. Ann Rheum Dis.

[2] Palazzo C, Nguyen C, Lefevre-Colau MM, Rannou F, Poiraudeau S (2016). Risk factors and burden of osteoarthritis. Ann Phys Rehabil Med.

[3] Litwic A, Edwards MH, Dennison EM, Cooper C (2013). Epidemiology and burden of osteoarthritis. Br Med Bull.

[4] World Health Organization. Defining Sexual Health. Geneva: WHO; 2006.

[5] Abdel-Nasser AM, Ali EI (2006). Determinants of sexual disability and dissatisfaction in female patients with rheumatoid arthritis. Clin Rheumatol.

[6] El Miedany Y, El Gaafary M, El Aroussy N, Youssef S, Ahmed I (2012). Sexual dysfunction in rheumatoid arthritis patients: arthritis and beyond. Clin Rheumatol.

[7] Josefsson KA, Gard G (2010). Women's experiences of sexual health when living with rheumatoid arthritis--an explorative qualitative study. BMC Musculoskelet Disord.

[8] Josefsson KA, Gard G (2012). Sexual health in patients with rheumatoid arthritis: experiences, needs and communication with health care professionals. Musculoskelet Care..

[9] Orzua-de la Fuente WM, Salazar-Hernandez GJ, Vega-Morales D, Garza-Alpirez A, Esquivel-Valerio JA (2018). High prevalence of sexual dysfunction in women with rheumatic diseases: a not recognized health domain. Sex Disabil.

[10] Batmaz I, Sariyildiz MA, Dilek B, Inanir A, Demircan Z, Hatipoglu N (2013). Sexuality of men with fibromyalgia: what are the factors that cause sexual dysfunction?. Rheumatol Int.

[11] Besiroglu MDH, Dursun MDM. The association between fibromyalgia and female sexual dysfunction: a systematic review and meta-analysis of observational studies. J Sex Med. 2019;31:288–97. 10.1038/s41443-018-0098-3 30467351

[12] Schairer LC, Foley FW, Zemon V, Tyry T, Campagnolo D, Marrie RA (2014). The impact of sexual dysfunction on health-related quality of life in people with multiple sclerosis. Mult Scler.

[13] Puchner R, Sautner J, Gruber J, Bragagna E, Trenkler A, Lang G (2019). High burden of sexual dysfunction in female patients with rheumatoid arthritis: results of a Cross-sectional study. J Rheumatol.

[14] Alia F, Rim B, Miladi S, Ouenniche K, Kassab S, Chekili S (2019). Comparison of sexual function in Tunisian women with rheumatoid arthritis and healthy controls. Clin Rheumatol.

[15] Restoux LJ, Dasariraju SR, Ackerman IN, Van Doornum S, Romero L, Briggs AM (2020). Systematic review of the impact of inflammatory arthritis on intimate relationships and sexual function. Arthritis Care Res.

[16] Dyer K, das Nair R (2013). Why don't healthcare professionals talk about sex? A systematic review of recent qualitative studies conducted in the United Kingdom. J Sex Med.

[17] Dahm DL, Jacofsky D, Lewallen DG (2004). Surgeons rarely discuss sexual activity with patients after THA - a survey of members of the American Association of hip and Knee Surgeons. Clin Orthop Relat Res.

[18] Harmsen RTE, den Oudsten BL, Putter H, Leichtenberg CS, Elzevier HW, Nelissen R (2018). Patient expectations of sexual activity after Total hip arthroplasty: a prospective multicenter cohort study. JBJS Open Access.

[19] Lavernia CJ, Villa JM (2016). High rates of interest in sex in patients with hip arthritis. Clin Orthop Relat Res.

[20] Meiri R, Rosenbaum TY, Kalichman L (2014). Sexual function before and after Total hip replacement: narrative review. Sex Med.

[21] Kazarian GS, Lonner JH, Hozack WJ, Woodward L, Chen AF (2017). Improvements in sexual activity after Total knee Arthroplasty. J Arthroplast.

[22] Wallis JA, Taylor NF, Bunzli S, Shields N (2019). Experience of living with knee osteoarthritis: a systematic review of qualitative studies. BMJ Open.

[23] Purdy R, Lister S, Salter C, Fleetcroft R, Conaghan P, Smith TO (2014). Living with osteoarthritis: a systematic review and meta-ethnography. Scand J Rheumatol.

[24] Tollefsrud I, Mengshoel AM. A fragile normality - illness experiences of working-age individuals with osteoarthritis in knees or hips. Disabil Rehabil. 2019:1–7. 10.1080/09638288.2019.1573930. 10.1080/09638288.2019.1573930 30829074

[25] Patton MQ (2015). Qualitative research & evaluation methods.

[26] Thorstensson CA, Garellick G, Rystedt H, Dahlberg LE (2015). Better Management of Patients with osteoarthritis: development and Nationwide implementation of an evidence-based supported osteoarthritis self-management Programme. Musculoskelet Care..

[27] Tong A, Sainsbury P, Craig J (2007). Consolidated criteria for reporting qualitative research (COREQ): a 32-item checklist for interviews and focus groups. Int J Qual Health Care.

[28] Ambler N, Williams AC, Hill P, Gunary R, Cratchley G (2001). Sexual difficulties of chronic pain patients. Clin J Pain.

[29] Ostlund G, Bjork M, Valtersson E, Sverker A (2015). Lived experiences of sex life difficulties in men and women with early RA - the Swedish TIRA project. Musculoskelet Care.

[30] Helland Y, Dagfinrud H, Haugen MI, Kjeken I, Zangi HD (2017). Patients' perspectives on information and communication about sexual and relational issues in rheumatology health care. Musculoskelet Care..

[31] Flynn KE, Lin L, Bruner DW, Cyranowski JM, Hahn EA, Jeffery DD (2016). Sexual satisfaction and the importance of sexual health to quality of life throughout the life course of US adults. J Sex Med.

[32] van Doornum S, Ackerman IN, Briggs AM (2019). Sexual dysfunction: an often overlooked concern for people with inflammatory. Expert Rev Clin Immunol.

[33] Traumer L, Jacobsen MH, Laursen BS (2019). Patients’ experiences of sexuality as a taboo subject in the Danish healthcare system: a qualitative interview study. Scand J Caring Sci.

[34] McInnes RA (2003). Chronic illness and sexuality. Med J Aust.

[35] Areskoug-Josefsson K, Gard G (2015). Physiotherapy as a promoter of sexual health. Physiother Theory Pract.

[36] Areskoug-Josefsson K, Gard G (2015). Sexual health as part of physiotherapy: the voices of physiotherapy students. Sex Disabil.

[37] Novick G (2008). Is there a bias against telephone interviews in qualitative research?. Res Nurs Health.

