# Supplementary material for: Experiences of sexual health in persons with hip and knee osteoarthritis: a qualitative study
Source: BMC Musculoskelet Disord. 2020 Aug 24;21:576. doi: 10.1186/s12891-020-03596-5 (PMC7445899; doi:10.1186/s12891-020-03596-5)
Supplement: Supplementary file 1 — Additional file 1. Interview guide. [file 12891_2020_3596_MOESM1_ESM.docx]

**Interview guide**

*Introduction*

- How has your daily life been affected by your hip or knee OA?

*Sexual health*

- Will you tell me about your relationship?

- Has your relationship been affected by your hip or knee OA? If so, how?

- How do you experience your intimate relationship?

- Has your intimate relationship been affected by your hip or knee OA? If so, will you tell me how?

*Communication*

- What effect has the physiotherapy had for you?

- Has issues regarding sexual health or your intimate relationship been raised with your physiotherapist? Will you tell me more about it?

- How do you perceive physiotherapists’ role in discussing sexual health?

- How would you suggest information on sexual health in OA could be provided?
